# Mississippi

**Published:** 1897-08

**Authors:** 


*Mississippi.*—Dr. Joseph C. Robert, of the Agricultural College, has been granted an appropriation of $2500 for the purpose of investigating the possibilities of preparing a vaccine or antitoxin to be used on Northern cattle to protect them from the dangers of Texas fever when purchased for the Southern fields, with the purpose in view of improving the live-stock of the South. A carload of cattle were recently purchased in St. Louis, Mo., to commence the line of experiments planned for.

---

The Francisco Dairy Co., of Newark, N. J., will erect a suitable building for the testing of all new animals introduced into their dairy; also to be used for the treating of all sick and injured animals.

